# Modulatory effects on dendritic cells by human herpesvirus 6

**DOI:** 10.3389/fmicb.2015.00388

**Published:** 2015-04-30

**Authors:** Rasmus Gustafsson, Mattias Svensson, Anna Fogdell-Hahn

**Affiliations:** ^1^Department of Molecular Medicine and Surgery, Karolinska Institutet, Karolinska University HospitalStockholm, Sweden; ^2^Department of Medicine, Center for Infectious Medicine, Karolinska Institutet, Karolinska University HospitalStockholm, Sweden; ^3^Department of Clinical Neuroscience, Karolinska Institutet, Karolinska University HospitalStockholm, Sweden

**Keywords:** HHV-6, dendritic cells, activation, cytokines, HLA, co-stimulatory molecules

## Abstract

Human herpesvirus 6A and 6B are β-herpesviruses approaching 100% seroprevalance worldwide. These viruses are involved in several clinical syndromes and have important immunomodulatory effects. Dendritic cells (DC) are key players in innate and adaptive immunity. Accordingly, DC are implicated in the pathogenesis of many human diseases, including infections. In this review the effects of HHV-6 infection on DC will be discussed.

## Introduction

Human herpesvirus 6 (HHV-6) is a ubiquitous β-herpes virus that most people have been exposed to by the age of 2 years (Okuno et al., 1989; Ward et al., 1993; Hall et al., 1994; Zerr et al., 2005). It was first isolated in the mid 80's from patients with lymphoproliferative disorders and acquired immunodeficiency syndrome (Salahuddin et al., 1986; Downing et al., 1987; Tedder et al., 1987). HHV-6 isolates are classified into two distinct virus species, HHV-6A and 6B (Ablashi et al., 2014). This review discusses the effect of HHV-6 on dendritic cells (DC).

The primary infection of HHV-6 usually gives mild fever and in a limited number of cases also a rash (Pruksananonda et al., 1992). The infection typically appear from 6 months of age, when the immune protection from the mother vanishes and before the immune system of the child is fully established (Stone et al., 2014). However, the virus can also cause fatal encephalitis as primary infection (Yamamoto et al., 2014), but typically this occurs only in immuno compromised patients due to, for example, induced immunosuppression after transplantation (Zerr et al., 2012). As an opportunistic infection the virus has been detected in different organs like heart (Hakacova et al., 2013; Escher et al., 2015) and tyroidea (Caselli et al., 2012). The reactions seen during the HHV-6 associated drug-reaction with eosinophilia and systemic symptoms syndrome show that reactivation of the virus can occur from multiple tissues and organs (Cacoub et al., 2011). There are also indications of involvement in CNS diseases like multiple sclerosis (Virtanen and Jacobson, 2012) and epilepsy (Theodore et al., 2008; Esposito et al., 2014), although these results have been difficult to verify in cross-sectional studies (Gustafsson et al., 2013a; Engdahl et al., 2014).

The viruses may have tropism for many different cell types, including immune cells. A predominant tropism is seen for CD4+ T cells (Lusso et al., 1988; Takahashi et al., 1989), but also CD8+ T cells (Lusso et al., 1991; Grivel et al., 2003), NK cells (Lusso et al., 1993), monocytes (Kondo et al., 1991; Niiya et al., 2006), macrophages (Kondo et al., 1991), and DC (Asada et al., 1999; Hirata et al., 2001; Kakimoto et al., 2002; Niiya et al., 2004; Takemoto et al., 2009). However, the role of HHV-6 interactions with host DC in the pathogenic events of HHV-6 infection remains unclear.

## Dendritic cells

DC are a heterogeneous family of cells with specialized antigen presenting capacities (Banchereau and Steinman, 1998; Schlitzer and Ginhoux, 2014). They can engulf extracellular material such as bacteria and virions, subsequently degraded in lysosomes. DC respond to microbial stimuli, such as pathogen-associated molecular patterns that are bound by pattern recognition receptors such as toll-like receptors (TLR) (Kaisho and Akira, 2006). DC also respond to inflammatory stimuli, such as inflammatory cytokines. Microbial and inflammatory stimuli can initiate a process of cellular activation termed maturation. The process of maturation is associated with increased surface levels of MHC and co-stimulatory molecules and other surface markers such as CD83, as well as enhanced production of soluble inflammatory mediators such as type I IFN, interleukin (IL)-8, IL-6, tumor necrosis factor (TNF), and IL-12 (Ueno et al., 2007).

In the orchestration of T cells, DC interact with the TCR on the T cells using its major histocompatibility complex (MHC) molecules that binds antigenic peptides. The co-stimulatory molecules CD80, CD86, and CD40 on the DC further strengthen the interactions by binding to their ligands CD28 and CD40L on the T cells. Upon such interaction, a so called “immunological synapse” is formed and signals are transmitted to the T cells (Huppa and Davis, 2003). Generally, MHC class I binds endogenous antigens (peptides) synthesized in the cells while MHC class II binds exogenous antigens engulfed by endocytic processes. Exogenous peptides are also presented on MHC-I in a process called cross-presentation (Van Montfoort et al., 2014). While DC together with B cells and macrophages, have the ability to express both MHC class I and class II molecules and consequently interact with both CD4+ T cells and CD8+ T cells, most other cells only express MHC class I. Viruses can hijack the replication machinery of host cells, and hence, the virus-infected cell displays viral peptides on its MHC class I molecules to allow interaction with CD8+ T cells leading to killing of the infected cells. In humans, MHC is termed human leukocyte antigen (HLA).

The two major sub groups of DC are plasmacytoid (pDC) and classical (cDC), and the cDC refer to all DC other than pDC (Merad et al., 2013). Pre-cDCs derive from common DC progenitors and migrate from the bone marrow via the blood and home to lymphoid and non-lymphoid tissues (Liu et al., 2009). Also, monocytes can differentiate into cDCs *in vivo* mainly in infected or inflamed tissues, and therefore monocytes have been viewed as precursors of inflammatory DC (Naik et al., 2006). Recent studies, however, have established that monocytes contribute to intestinal (Bogunovic et al., 2009; Varol et al., 2009), splenic (Lewis et al., 2011), and muscular cDC also at steady state (Langlet et al., 2012).

Although *in vitro* culturing of human CD34+ progenitors with the Flt3L and thrombopoietin has proven useful in generating both pDC and cDC (Chen et al., 2004; Poulin et al., 2010), the dominant model for the generation of human DC has involved culturing monocytes with colony-stimulating factor 2 (CSF-2) and IL-4, which primarily produces dermal-like CD1a+ cDC (Sallusto and Lanzavecchia, 1994; Palucka et al., 1998). CSF-2-stimulated cultures reliably produce large numbers of monocyte-derived DC (MDC), which is invaluable when designing experiments to study the functional consequences of pathogen interactions with host DC in human infections.

## Virus replication

The vast majority of studies investigating HHV-6A or 6B infection in DC have studied MDC. The literature provides contradictory reports on the replication capacity of HHV-6A (Hirata et al., 2001; Smith et al., 2005; Gustafsson et al., 2013b) (Table 1). Whereas, Hirata et al. (2001) suggests that DC can support HHV-6A (U1102 strain) replication by increase in protein load over time, Smith et al. (2005) suggests that DC cannot support HHV-6A (GS strain) replication as no increase in intracellular viral DNA was seen over time. We did not detect any increase in either viral protein or DNA when infecting DC with HHV-6A (GS strain) (Gustafsson et al., 2013b), thus supporting the report by Smith et al. To control for degraded virus or suboptimal infections both Smith et al. and we infected susceptible cells in parallel with DC using the same virus supernatants. Productive infection was seen in the susceptible cells suggesting that lack of viral replication seen in DC was not due to problems with the virus stock or infection procedure. Hence, the discordant results might lay in other factors such as the different viral strains or passages used. Whereas U1102 strain showed productive infection, GS strain did not.

**Table 1:** **Virus propagation procedures, virus replication assessment and surface expression of relevant molecules upon HHV-6 exposure of MDC**.

**First author**	**Year**	**HHV-6 spieces**	**Strains**	**Cells for prop**.	**Ultracent. of inoc**.	**MOI**	**Virus repl**.	**HLA-I**	**HLA-II**	**CD40**	**CD44**	**CD80**	**CD83**	**CD86**	**DC-SIGN**
Asada	1999	6B, 6A ns	Z29	Un	Yes	0.1	Yes 6B								
Hirata	2001	6A and 6B	U1102/HST	CBMC	No	0.1	Yes	− 6A (im)	± 6A (im)				± 6A (im)		
								± 6B (im)	± 6A (im)				± 6B (im)		
Kakimoto	2002	6A and 6B	Z29/U1102	CBMC	No	1	Yes	+ (im)	+ (im)	± (im)	− (im)	+ (im)	+ (im)	+ (im)	
		(6A ns)													
Niiya	2004	6A and 6B	U1102/HST	Un	Un	1	Ns					+ (im)	+ (im)	+ (im)	− (im)
		(6A ns)													
Smith	2005	6A and 6B	GS/PL-1	CBMC	Yes	1	No	+ 6A (im)	+ 6A (im)	± 6A (im)		± 6A (im)			
								− 6A (mat)	+ 6B (mat)	− 6A (mat)		− 6A (mat)			
Niiya	2006	6A and 6B	U1102/Z29	CBMC	Un	1	Yes	± (im)	+ (im)	± (im)	± (im)	+ (im)	± (im)	+ (mat)	− (im)
		(6A ns)						± (mat)	+ (mat)	± (mat)	± (mat)	± (mat)	± (mat)	+ (im)	
Takemoto	2009	6B	HST	CBMC	No	1	Partial							+ (im)	
Bertelsen	2010	6B	PL-1	Molt-3	Yes	2.5 e−4	Unclear		+ (im)	+ (im)		+ (im)	+ (im)	+ (mat)	
									± (mat)	− (mat)		− (mat)	± (mat)	+ (im)	
Murakami	2010	6B	Z29	Un	Un	1	Yes								
Gustafsson	2013	6A	GS	HSB-2	No	0.01	No	+ (im)	+ (im)	± (im)			± (im)	+ (im)	

***Abbreviations used:** −, downregulation upon virus exposure; +, upregulation upon virus exposure; ±, unaltered expression/secretion/effets upon virus exposure; mat, mature DC; im, immature DC; Un, unknown; ns, not shown; 6A, HHV-6A; 6B, HHV-6B. Blank boxes indicate that no measurment was shown*.

For HHV-6B, the literature is more consistent. This is seen by different read-outs including increased virus titers in supernatants over time (Asada et al., 1999), detection of virus proteins using immunofluoresence staining (Asada et al., 1999; Hirata et al., 2001) or flow cytometry; and detection of viral mRNA expression (Kakimoto et al., 2002; Niiya et al., 2006; Takemoto et al., 2009). Assessing the presence of viral proteins is somewhat problematic given the difficulty to discriminate between viral proteins from the inoculum or from *de novo* virus synthesis, as it is not feasible to completely wash away virus particles after inoculation. An alternative approach is to assess shifts in levels of viral DNA (Asada et al., 1999). Translation of early and late HHV-6B genes has been seen, however, Takemoto et al. (2009) detected few virions and thereby conclude that replication in DC might be incomplete. However, transmission to allogenic matured CD4+ T cells upon co-culture was seen, and conversion to productive replication via cell-to-cell contact (Takemoto et al., 2009). This was not seen for HHV-6A (Gustafsson et al., 2013b). In conclusion, DC seem to be able to support a productive infection of HHV-6B and transmit the virus to T cells, constituting an important vector for transporting the virus to the most susceptible cell type. For HHV-6A, more studies are needed and the positive result shown should be repeated using other viral strains or clinical isolates to provide more robust proof of tropism.

## Immune modulation

### HLA

Among the various aspects of immunomodulation, HHV-6 has been suggested to impair differentiation of monocytes to DC (Niiya et al., 2006). The effects on DC, however, remain controversial (Table 1). Whereas some studies report up-regulation of HLA class I on immature DC upon exposure to HHV-6A or 6B, both with or without virus replication (Kakimoto et al., 2002; Smith et al., 2005; Gustafsson et al., 2013b) others have shown virus replication-dependent down-regulation for HHV-6A but not for 6B (Hirata et al., 2001), and some have shown no effect at all (Niiya et al., 2006). In our hands HLA class I expression was induced by autocrine IFN-α signaling (Gustafsson et al., 2013b). Two studies have investigated the expression on matured DC and demonstrated down-regulation or unaltered expression (Smith et al., 2005; Niiya et al., 2006). These differences are explained neither by the cell type used for virus propagation nor by whether ultracentrifugation on virus inoculum was used or not. Likewise, presence or absence of virus replication cannot explain the conflicting results. Another drawback in comparisons with older studies is that sometimes the specific virus species used for a certain experiment is not described, probably as HHV-6A and HHV-6B was recognized as two separate viruses as late as in 2014. The same inconsistency is seen for HLA class II upon HHV-6A and HHV-6B infection where some studies report up-regulation; some report down-regulation and some show an unaltered expression (Hirata et al., 2001; Kakimoto et al., 2002; Smith et al., 2005; Niiya et al., 2006; Bertelsen et al., 2010; Gustafsson et al., 2013b). To conclude, despite the elegant pioneering study by Hirata et al. that convincingly showed a HHV-6A exclusive suppressive effect on HLA class I, the effects on HLA I/II expression does not seem to be very strong given the varying results in subsequent studies.

### Co-stimulatory molecules and other surface markers

Regarding co-stimulatory molecules a vast majority of studies report conclusive data for up-regulation of CD86 and unaltered expression of CD40 on immature DC (Kakimoto et al., 2002; Smith et al., 2005; Niiya et al., 2006; Gustafsson et al., 2013b), regardless of virus replication. Also for mature DC, CD86 is up-regulated whereas CD40 expression is suppressed by both HHV-6A and 6B (Smith et al., 2005; Bertelsen et al., 2010). For CD80 a productive virus replication of HHV-6B seems to lead to up-regulation in immature DC (Kakimoto et al., 2002; Niiya et al., 2004; Bertelsen et al., 2010), whereas mature DC down-regulate CD80 upon exposure with or without replication for both HHV-6A and 6B (Smith et al., 2005; Bertelsen et al., 2010). CD83 is unaltered or up-regulated upon infection with both viruses and on both immature and matured DC. Hence, independent of productive replication, neither HHV-6A nor 6B seems to suppress the functions of DC on the level of co-stimulatory molecules but rather induce maturation and activation.

Dendritic Cell-Specific Intercellular adhesion molecule-3-Grabbing Non-integrin (DC-SIGN) and CD44 are cell surface molecules that are important for DC homing and T cell maturation. One study suggests that DC-SIGN is down-regulated by replicating HHV-6A and 6B, as UV inactivation of the virus prior to inoculation abrogated the suppressive effect (Niiya et al., 2004). Similarly, CD44 was suppressed or unaltered on DC upon HHV-6 infection (Kakimoto et al., 2002; Niiya et al., 2006). Thus, HHV-6 might have suppressive effects on the ability of DC to bind tissues such as endothelium.

### Cytokines

Interferon (IFN) type I and III are important mediators of antiviral immunity, secreted upon virus infection, and they have a range of different effects. Interferon-α and -λ both signal via the Janus kinase—signal transducers and activators of transcription pathway but they utilize different cell surface receptors (Sheppard et al., 2003; Gibbert et al., 2013). Whereas HHV-6B exposure can induce both IFN-α and -λ secretion by pDC (Nordstrom and Eriksson, 2012), HHV-6A exposure induces strong IFN-α, but low IFN-λ secretion by MDC (Gustafsson et al., 2013b) (Table 2).

**Table 2:** **Cytokine secretion upon HHV-6 exposure**.

**First author**	**Year**	**DC type**	**HHV-6 spieces**	**IFN**	**IL-6**	**IL-8**	**IL-10**	**IL-12**	**TNF**	**MIP-1b**	**RANTES**
Smith	2005	MDC	6A and 6B				± 6A (mat)	− 6A (im)	± 6A (mat)	± 6A (mat)	± 6A (mat)
								− 6B (im)			
Takemoto	2009	MDC	6B		+ (im)	+ (im)		+ (im)	+ (im)		
Bertelsen	2010	MDC	6B				+ (mat)	+ (mat)			
Murakami	2010	MDC	6B		± (im)	± (im)	± (im)				
					− (mat)	− (mat)	− (mat)				
Nordström	2012	pDC	6B	+ IFN-a (im)							
				+ IFN-g (im)							
				+ IFN-l (im)							
Gustafsson	2013	MDC	6A	+ IFNa (im)	± (im)	− (im)	+ (mat)	± (im)			
				± IFN-l (im)	± (mat)	+ (mat)		+ (mat)			

***Abbreviations used:** −, downregulation upon virus exposure; +, upregulation upon virus exposure; ±, unaltered expression/secretion/effets upon virus exposure; mat, mature DC; im, immature DC; 6A, HHV-6A; 6B, HHV-6B. Blank boxes indicate that no measurment was shown*.

IL-12 is involved in the differentiation of T cells toward a Th1 lineage. Suppression of IL-12 secretion upon non-productive infection of both HHV-6A and 6B has been shown to occur both in DC (Smith et al., 2005) and macrophages (Smith et al., 2003). The opposite effect in DC with augmented levels has been seen for both HHV-6A (Gustafsson et al., 2013b) and HHV-6B (Bertelsen et al., 2010). Reasons for this divergence is unclear but at least does not seem to lie in the procedures used for virus propagation as the two studies demonstrating augmented secretion used crude supernatant or ultracentrifuged virus. The data on IL-12 described above was generated on mature DC. In immature DC the secretions are much more modest with a slight increase (Takemoto et al., 2009) or undetectable secretion (Smith et al., 2005; Gustafsson et al., 2013b). Possibly, therefore the role of IL-12 may not be of major importance for immunity against HHV-6, but this needs to be investigated further.

TNF secretion can be induced by IL-12 (Nagayama et al., 2000). Three studies have looked at TNF secretion and report conflicting results that however, at least in part, can be explained by the IL-12 profiles. Whereas the single study that report modest increased secretion of TNF by immature DC upon productive HHV-6B infection also see increased IL-12 secretion (Takemoto et al., 2009), we and others saw unaltered and nearly undetectable levels of TNF, along with IL-12 upon non-productive HHV-6A infection, as described above (Smith et al., 2005; Gustafsson et al., 2013b). Hence, the different results might be explained by the use of different viruses and/or virus replication. In matured DC, however, the levels of TNF are more dramatic. In line with the IL-12 profiles described above we see augmented secretion of TNF (Gustafsson et al., 2013b) whereas Smith et al. (2005) see unaltered or slightly decreased secretion.

IL-6 secretion is important in acute phases of inflammation. Whereas immature DC slightly up-regulated IL-6 secretion upon HHV-6B infection and replication (Takemoto et al., 2009), mature DC down-regulated secretion, due to impaired TLR4 signaling (Murakami et al., 2010). For HHV-6A minute levels were secreted by immature DC, and for mature DC the virus somewhat augmented the secretion (Gustafsson et al., 2013b). Therefore, HHV-6B might have an exclusive ability to suppress TLR4 signaling that is not seen for HHV-6A, in terms of IL-6 secretion, but this potential difference between the virus strains remains to be tested.

IL-8 is a crucial cytokine which induces migration of neutrophils across vascular walls (Atta Ur et al., 1999). HHV-6A has been shown to strongly suppress IL-8 secretion in immature DC, but this effect was overridden when other stimuli such as lipopolysaccharides and IFN-γ was present (Gustafsson et al., 2013b). HHV-6B displays a mirror image with slightly enhanced secretion by immature DC (Takemoto et al., 2009) but suppressed secretion by mature DC (Murakami et al., 2010). As for IL-6, described above, an exclusive ability of HHV-6B to suppress TLR4 signaling might explain these differences.

The ability to control immune responses to restore tissue homeostasis is crucial for the organism. IL-10 has anti-inflammatory properties and suppresses secretion of inflammatory cytokine and expression of HLA class II and co-stimulatory molecules. Whereas HHV-6A inoculation does not affect IL-10 secretion in mature DC (Smith et al., 2005), inconsistent data for HHV-6B has been published with down- or up-regulation (Bertelsen et al., 2010; Murakami et al., 2010). Hence, additional studies are needed to clarify the role of IL-10 in HHV-6 infection.

### T cell stimulation

The capacities of DC to stimulate T cell expansion is crucial and the literature is quite consistent with four out of five studies showing a reduced allostimulatory capacity upon inoculation (Asada et al., 1999; Kakimoto et al., 2002; Smith et al., 2005; Niiya et al., 2006; Gustafsson et al., 2013b) (Table 3). This effect is seen for HHV-6A as well as for HHV-6B, regardless of virus replication, and therefor the evidence is strong. The two studies assessing the effect on autologous T cell activation both observed an impaired capacity upon inoculation with both HHV-6A and 6B (Kakimoto et al., 2002; Niiya et al., 2006). This effect was also seen *in vivo*, as demonstrated by an elegant experiment where allogenic T cells were co-cultured with MDC from a patients with drug induced hypersensitivity syndrome during HHV-6 viremia and recovery phases. The allostimulatory capacity was clearly reduced during viremia (Niiya et al., 2006). HHV-6 also seems to possess more indirect suppressive effects by the induction of virus specific Tregs which can accomplish a suppression of the maturation of MDC *in vivo* (Wang et al., 2014).

**Table 3:** **Studies on allo- and autoreactivity after HHV-6 exposure of MDC**.

**First author**	**Year**	**HHV-6 spieces**	**Allostimulation**	**Autostimulation**
Asada	1999	6A and 6B (6A ns)	± 6B (im)	
Kakimoto	2002	6A and 6B (6A ns)	− (im)	− (im)
Smith	2005	6A and 6B	− 6A (mat)	
			− 6A (im)	
Niiya	2006	6A and 6B	− 6A (im)	− (im)
			− 6B (im)	
Gustafsson	2013	6A	− (im)	

***Abbreviations used:** −, downregulation upon virus exposure; +, upregulation upon virus exposure; ±, unaltered expression/secretion/effets upon virus exposure; mat, mature DC; im, immature DC; ns, not shown; 6A, HHV-6A; 6B, HHV-6B. Blank boxes indicate that no measurment was shown*.

## Conclusions and future perspectives

Altered cytokine secretion profiles, suppressed expression of co-stimulatory molecules and HLA molecules and cytopathic effects in response to HHV-6 may critically impact HHV-6 pathogenesis. The ability of DC to activate and induce proliferation of T cells, as well as establishing appropriate cytokine milieus required for T cell polarization makes further studies on mechanisms underlying the suppressive effects seen by HHV-6 necessary. The literature provides consistent results on the suppressive effect of HHV-6 on T cell proliferation. In contrast, reports on the effects on cytokine secretion and surface expression of co-stimulatory and HLA molecules on DC are contradictory. Therefore, for the impairment of T cell proliferation, cytokines, or surface molecules may not have a major impact but may result from other factors. However, to test impacts of soluble factors, conditioned media from virus infected DC (filtered to remove virions) can be used to treat co-cultures with DC and T cells. To substantiate the findings reported so far the data should be repeated with better standardization of virus propagation procedures and comparisons between different virus strains or clinical isolates to increase the concordance between different studies.

### Conflict of interest statement

The authors declare that the research was conducted in the absence of any commercial or financial relationships that could be construed as a potential conflict of interest.
